# Supplementary material for: The Combined Effects of Dietary Diversity and Frailty on Mortality in Older Taiwanese People
Source: Nutrients. 2022 Sep 16;14(18):3825. doi: 10.3390/nu14183825 (PMC9502423; doi:10.3390/nu14183825)
Supplement: Supplementary file 1 [file nutrients-14-03825-s001.zip › nutrients-1870536-supplementary.pdf]

Table S1. The definition of a serving for six food groups.

Food groups	Definition of one serving
Grain	15 g of carbohydrate
Soybeans/fish/eggs/meat	7 g of protein
Dairy	8 g of protein
Oil/fat/nuts	5 g of fat
Vegetables	25 kcal
Fruit	60 kcal

Table S2. Baseline characteristics by frailty severity among NAHSIT 2005–2008 older adults (*n* = 968).

	Frailty severity			
	Robust	Prefrailty	Frailty	P-value
n, %	582 (60.3)	349 (35.8)	37 (3.9)	
Weight sample size (n)	1,372,246	815,767	88,343	
Gender, %				0.004
Men	53.9	42.3	46.9	
Women	46.1	57.7	53.2	
Age (years), %	72.8 ± 0.34	74.5 ± 0.40	75.2 ± 1.10	0.003
65–69	36.2	26.6	23.4	0.006
70–74	25.9	26.1	18.1	
75–79	24.4	27.0	33.0	
≥ 80	13.6	20.3	25.5	
Education, %				0.074
Illiterate	24.4	29.7	38.7	
Some up to primary school	57.1	50.7	54.0	
High school and above	18.6	19.6	7.30	
Smoking, %	35.3	31.4	41.9	0.376
Drink alcohol, %	33.7	27.7	42.9	0.171
Enough money, %				0.668
More than enough	6.15	7.95	5.98	
Just enough	63.3	57.7	48.0	
difficult	30.6	34.4	46.0	
Perceived health status, %				<0.001
Good	34.3	22.3	6.53	
Fair	54.3	44.9	21.5	
Poor	11.4	32.9	72.0	
Poor sleep quality, %	6.74	13.0	20.4	<0.001
Number of diseases	1.85 ± 0.06	3.55 ± 0.15	5.69 ± 0.37	<0.001
Number of drug treatments	0.92 ± 0.05	1.91 ± 0.10	3.71 ± 0.26	<0.001
Cognitive impairment[¶], %	13.3	19.0	20.6	0.125
Supplement use, %	42.8	58.8	48.2	0.003
BMI (kg/m^2)	24.5 ± 0.19	24.8 ± 0.40	25.0 ± 1.22	0.412
Waist circumference (cm)	86.0 ± 0.51	87.4 ± 0.97	86.7 ± 3.03	0.429
Hip circumference (cm)	93.2 ± 0.53	94.0 ± 0.88	93.1 ± 2.64	0.357

Data were weighted for unequal probability of sampling design by SUDAAN software.

Categorical variables are presented as percent, and continuous variables are presented as mean ± SEM.

One-way analysis of variance (ANOVA) and chi-square test were used for continuous and categorical variables.

Cognitive impairment¶ was defined by an SPMSQ with ≥3 errors.

Table S3. Baseline characteristics by frailty severity among NAHSIT 2005–2008 older men (*n* = 482).

	Frailty severity			
	**Robust**	**Prefrailty**	**Frailty**	**P-value**
n, %	316	149	17	
Weight sample size (n)	739,492	344,817	41,386	
Age (years), %	73.0 ± 0.35	74.7 ± 0.59	75.6 ± 1.72	0.008
65–69	32.3	27.3	24.2	0.163
70–74	26.5	21.7	21.5	
75–79	28.6	30.4	17.1	
≥80	12.1	20.6	37.2	
Education, %				0.012
Illiterate	7.93	10.7	21.0	
Up to primary school	61.8	50.2	76.6	
High school and above	30.3	39.1	2.36	
Smoking, %	61.9	67.9	81.0	0.178
Drink alcohol, %	52.0	54.9	55.4	0.850
Enough money, %				0.641
More than enough	7.72	11.0	4.65	
Just enough	63.2	57.4	57.5	
difficult	29.1	31.6	37.9	
Perceived health status, %				0.003
Good	40.0	29.1	13.8	
Fair	50.3	41.6	8.77	
Poor	9.70	29.4	77.4	
Poor sleep quality, %	3.65	14.9	8.52	<0.001
Number of diseases	1.92 ± 0.07	4.12 ± 0.21	5.66 ± 0.66	<0.001
Number of drug treatments	0.93 ± 0.06	2.02 ± 0.15	3.50 ± 0.41	<0.001
Cognitive impairment[¶], %	4.28	10.6	17.9	0.117
Supplement use, %	38.9	61.9	25.1	<0.001
BMI (kg/m^2)	24.2 ± 0.26	23.7 ± 0.59	22.6 ± 1.19	0.195
Waist circumference (cm)	87.2 ± 0.68	86.7 ± 1.62	86.2 ± 2.31	0.797
Hip circumference (cm)	92.5 ± 0.63	92.7 ± 1.26	90.8 ± 2.18	0.782

Data were weighted for unequal probability of sampling design by SUDAAN software.

Categorical variables are presented as percent, and continuous variables are presented as the mean ± standard error of the mean (SEM).

One-way ANOVA and chi-square test were used for continuous and categorical variables.

[¶]Cognitive impairment was defined by an SPMSQ with ≥3 errors.

Table S4. Baseline characteristics by frailty severity among NAHSIT 2005–2008 older women (n = 486).

	Frailty severity			
	Robust	Prefrailty	Frailty	P-value
n, %	266	200	20	
Weight sample size (n)	632,754	470,950	46,957	
Age (years), %	72.6 ± 0.57	74.3 ± 0.56	74.8 ± 1.29	0.075
65–69	40.0	26.0	22.6	0.001
70–74	25.2	29.3	15.0	
75–79	19.5	24.6	47.1	
≥80	15.3	20.2	15.3	
Education, %				0.618
Illiterate	43.6	43.7	54.4	
Up to primary school	51.5	51.1	34.0	
High school and above	4.97	5.28	11.7	
Smoking, %	2.54	5.10	7.45	0.305
Drink alcohol, %	11.3	7.93	31.9	0.091
Enough money, %				0.534
More than enough	4.23	5.66	7.16	
Just enough	63.3	58.0	39.6	
difficult	32.5	36.4	53.2	
Perceived health status, %				<0.001
Good	27.5	17.2	0.00	
Fair	59.0	42.4	32.8	
Poor	13.5	35.4	67.2	
Poor sleep quality, %	10.5	11.7	30.6	0.149
Number of diseases	1.77 ± 0.11	3.13 ± 0.16	5.71 ± 0.34	<0.001
Number of drug treatments	0.90 ± 0.07	1.83 ± 0.11	3.90 ± 0.37	<0.001
Cognitive impairment[¶], %	24.6	25.2	23.0	0.235
Supplement use, %	47.3	56.5	68.6	0.138
BMI (kg/m^2)	24.8 ± 0.23	25.7 ± 0.43	26.6 ± 1.64	0.045
Waist circumference (cm)	84.6 ± 0.80	87.2 ± 1.02	87.0 ± 5.00	0.013
Hip circumference (cm)	94.0 ± 0.60	95.1 ± 0.76	94.8 ± 4.42	0.323

Data were weighted for unequal probability of sampling design by SUDAAN software.

Categorical variables are presented as percent, and continuous variables are presented as the mean ± SEM.

One-way analysis of variance and chi-square test were used for continuous and categorical variables.

[¶]Cognitive impairment was defined by an SPMSQ with ≥3 errors.

Table S5. Distributions of food intake frequency by DDS among NAHSIT 2005–2008 older adults (n = 329).

	DDS			
	≤4	5	6	P for trend
n, %	124 (37.7)	138 (42.0)	67 (20.4)	
Food intake frequency, times/weekly				
Egg	2.09 ± 2.15	2.31 ± 2.17	3.31 ± 3.07	0.001
Milk and milk powder	1.74 ± 3.40	2.77 ± 3.89	6.22 ± 3.39	<0.001
Processed dairy products	0.30 ± 1.18	0.56 ± 1.48	1.28 ± 2.31	<0.001
Rice	15.5 ± 6.01	14.9 ± 5.15	13.7 ± 4.71	0.039
Noodles products	3.36 ± 3.42	4.00 ± 4.26	3.65 ± 3.24	0.450
Roots and tubers	1.41 ± 3.23	1.38 ± 2.58	2.43 ± 4.48	0.075
Breakfast cereals	1.32 ± 2.42	1.83 ± 3.91	3.67 ± 3.34	<0.001
Beans	0.55 ± 2.20	0.40 ± 1.02	0.40 ± 0.96	0.473
Vegetables	22.8 ± 14.3	24.9 ± 13.9	29.0 ± 21.0	0.013
Mushrooms	0.70 ± 1.17	0.99 ± 1.32	1.55 ± 1.94	<0.001
Pickled vegetables	1.08 ± 2.44	0.95 ± 2.85	0.59 ± 1.38	0.210
Fresh fruit	4.81 ± 5.08	8.54 ± 6.64	10.6 ± 6.18	<0.001
Processed fruit products	0.50 ± 1.63	0.58 ± 1.57	0.89 ± 2.38	0.164
Nuts and seeds	1.00 ± 2.57	1.28 ± 2.29	1.36 ± 2.59	0.291
Soybean products	3.31 ± 4.67	2.67 ± 2.83	3.49 ± 4.30	0.988
Seafood fish and shellfish	6.67 ± 6.08	6.90 ± 6.02	9.03 ± 7.51	0.030
Poultry	0.94 ± 1.28	1.39 ± 1.89	1.32 ± 1.67	0.064
Livestock	3.50 ± 4.74	4.11 ± 3.56	3.55 ± 3.83	0.740
Organ meats	0.12 ± 0.29	0.18 ± 0.68	0.29 ± 0.95	0.077
Blood and other parts	0.12 ± 0.31	0.24 ± 0.53	0.16 ± 0.31	0.281
Processed meat	0.91 ± 1.41	0.96 ± 1.06	1.08 ± 1.62	0.410
Sweetened beverages	0.33 ± 1.31	0.53 ± 2.48	0.27 ± 0.95	0.997
Tea	4.32 ± 8.34	3.30 ± 5.53	5.98 ± 9.80	0.304
Coffee	0.27 ± 1.08	0.79 ± 2.00	1.02 ± 2.70	0.006
Chocolate products	0.87 ± 2.45	0.91 ± 2.49	1.14 ± 4.64	0.580
Snacks	1.91 ± 2.6	2.66 ± 3.02	3.63 ± 4.40	<0.001

All variables are presented as the mean ± SD, and based on the general linear model regression.
